# Getting More Mileage from Lymph Node Biopsies

**DOI:** 10.1371/journal.pmed.0020324

**Published:** 2005-09-06

If breast cancer cells escape from the initial tumor site, they often travel to axillary lymph nodes (ALNs) in the armpit. Surgical removal and biopsy of axillary lymph nodes are routinely used to assess the stage of a patient's cancer, and prevent cancers that have spread into the lymph nodes from further metastasis. Because ALN dissections can cause uncomfortable chronic arm swelling, they are often preceded by a biopsy of the sentinel lymph node, the first node into which the tumor tissue drains. If sentinel lymph node biopsy reveals evidence of metastasis, surgeons then remove and dissect the ALNs. Lymph nodes are primarily examined for the presence of metastatic tumor cells, and, more recently, researchers are even searching for the presence of isolated tumor cells and breast cancer–associated gene expression patterns in local nodes.

In light of increasing evidence that the immune system is perturbed both locally at the tumor site and systemically as the cancer progresses, Peter Lee and colleagues set out to study the state of the immune system in draining lymph nodes. They determined profiles of CD4 and CD8 lymphocytes and dendritic cells from sentinel node biopsies and (nonsentinel) axillary lymph node dissections. Their results suggest that these immune profiles harbor independent information about the likelihood of tumor recurrence.

The researchers used automated high-resolution imaging to determine the numbers of CD4 T cells, CD8 T cells, and CD1a-positive dendritic cells in 47 sentinel and 104 axillary nodes from 77 patients with breast cancer. Five-year follow-up data were available for all patients, and 33 patients had disease recurrence within that time. The researchers found that sentinel and axillary nodes from cancer patients (whether the nodes contained tumor cells or were tumor free) contained fewer CD4 and CD8 T lymphocytes than nodes from cancer-free control patients. Dendritic cells were also reduced in tumor-positive nodes, but increased in tumor-free axillary nodes. [Fig pmed-0020324-g001]

**Figure pmed-0020324-g001:**
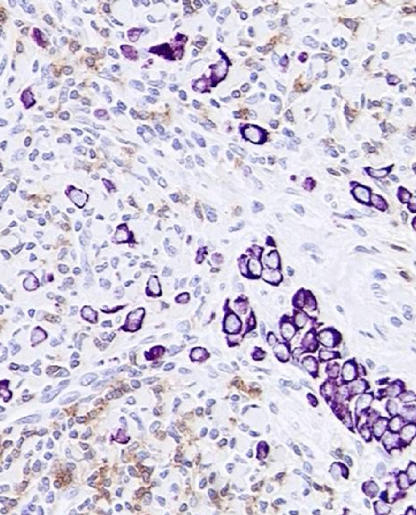
Sentinel lymph node section with immune cells and tumor cells

By dividing the patients into a “training set” of 29 individuals and a “test set” of 48 individuals, the researchers could test whether the correlations found in the test set could predict disease-free survival. They found that axillary node CD4 T cell and dendritic cell numbers, regardless of tumor status, were correlated with disease-free survival, but that this was not the case for immune parameters in the sentinel nodes (all of which were tumor-positive). Moreover, in their dataset, the predictive power of the immune parameters in the axillary nodes was better than that of any other characteristics of the patients, including pathological parameters such as tumor size and extent or size of nodal metastases.

These results suggest that, in patients with tumor-positive sentinel nodes, immune profile data from axillary nodes hold additional information on the probability of disease recurrence. As the authors suggest, these results warrant larger prospective studies to test these relationships and explore them in more detail. Another important open question is whether immune profile information from lymph nodes can predict risk of recurrence even in women whose cancers are caught at a stage where they have not yet spread to any lymph nodes.

